# Effect of dipeptidyl peptidase-4 inhibitors on cisplatin-induced acute nephrotoxicity in cancer patients with diabetes mellitus: A retrospective study

**DOI:** 10.1371/journal.pone.0229377

**Published:** 2020-02-21

**Authors:** Takamasa Iwakura, Hirotaka Fukasawa, Atsushi Kitamura, Kento Ishibuchi, Hideo Yasuda, Ryuichi Furuya

**Affiliations:** 1 Renal Division, Department of Internal Medicine, Iwata City Hospital, Iwata, Japan; 2 Internal Medicine I, Division of Nephrology, Hamamatsu University School of Medicine, Hamamatsu, Japan; Consiglio Nazionale delle Ricerche, ITALY

## Abstract

**Background:**

Cisplatin is a highly effective chemotherapeutic agent. However, acute kidney injury (AKI) limits its subsequent use, resulting in poor cancer prognosis. Dipeptidyl peptidase-4 (DPP-4) inhibitors have been reported to attenuate cisplatin-induced AKI in animal models, but the effect in human patients remains to be clarified. We hypothesized that DPP-4 inhibitors can prevent cisplatin-induced AKI in diabetic-cancer patients.

**Methods:**

We retrospectively reviewed all consecutive cancer patients who were treated with a first cycle of cisplatin-containing regimen between January 2011 and October 2019. We analysed data of diabetic-cancer patients treated with high-dose cisplatin (> 50 mg/m^2^)-containing regimens. The change of estimated glomerular filtration rate (eGFR) within 2 weeks after cisplatin treatment was compared between the patients treated with DPP-4 inhibitors and those treated without DPP-4 inhibitors.

**Results:**

A total of 455 patients were treated with cisplatin during the period. Of these, 34 patients were eligible for the analysis. The change of eGFR was significantly less in the patients treated with DPP-4 inhibitors, compared to those without DPP-4 inhibitors [the percentages of eGFR decline (mean ± SD) was 23.6 ± 20.3% vs 43.1± 20.1%, respectively; P = 0.010]. Furthermore, the incidence of AKI was significantly less in the patients treated with DPP-4 inhibitors (25% vs 64%, respectively; P = 0.026).

**Conclusions:**

DPP-4 inhibitors may decrease the risk of cisplatin-induced AKI in diabetic patients.

## Introduction

Cisplatin is one of the widely used chemotherapeutic agents for many types of malignancies, but frequently induces acute kidney injury (AKI). The adverse effect of AKI limits subsequent dosing, which deprives patients of an effective treatment for their malignancies [1]. Indeed, long-term survival of patients who experienced cisplatin-induced AKI was worse despite continuation of reduced dose of cisplatin afterward [2]. Hydration of saline, co-administration of mannitol and magnesium preloading are clinically used for the prevention of cisplatin-induced AKI [3], however, these preventive effects are still insufficient.

Dipeptidyl peptidase-4 (DPP-4) inhibitors, which are commonly used to control blood glucose levels, exert pleiotropic effects beyond its prescribed use for diabetic patients. Experimentally, DPP-4 inhibitors have been reported to attenuate cisplatin-induced AKI in mice and rats via inhibition of tubular cell death [4, 5]. It has also been reported that DPP-4 inhibitors can prevent AKI induced by ischemia-reperfusion and chronic kidney injury in several animal models [6–10]. However, it remains to be investigated whether DPP-4 inhibitors can attenuate kidney injury in human patients.

We hypothesized that DPP-4 inhibitors can attenuate acute phase of cisplatin-induced nephrotoxicity in human patients as same as rodent models. This study aims to compare the change of kidney function and the incidence of AKI in diabetic-cancer patients treated with cisplatin combined with or without DPP-4 inhibitors.

## Patients and methods

### Patients

**Fig 1:**
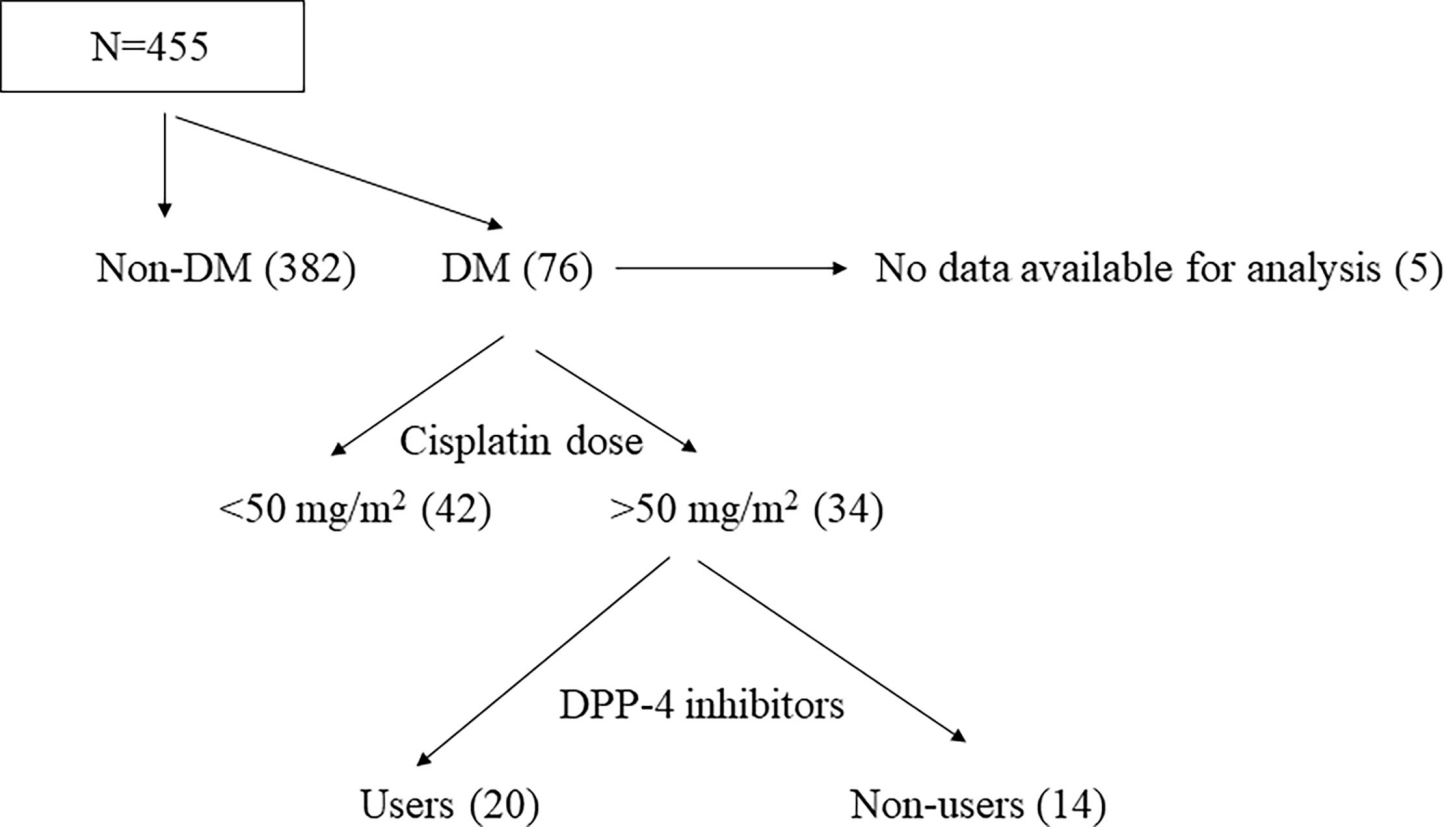
Flowchart demonstrating the inclusion process. Abbreviations: DM, diabetes mellitus. DPP-4, dipeptidyl peptidase-4.

We retrospectively reviewed all consecutive cancer patients who were treated with a first cycle of cisplatin-containing regimen between January 2011 and October 2019 at Iwata city hospital (Iwata, Japan). A total of 455 patients were treated with cisplatin during the period (Fig 1). Of these, 76 patients (16.7%) had diabetes mellitus. As the nephrotoxicity of cisplatin is dose-dependent [11], we included patients treated with high- dose cisplatin (> 50 mg/m^2^) for the analysis [12, 13]. To evaluate the effect of DPP-4 inhibitors on cisplatin-induced nephrotoxicity, patients were divided into 2 groups, users or non-users of DPP-4 inhibitors (DPP-4 inhibitor group and non-DPP-4 inhibitor group, respectively). This study was approved by the ethics committee of the Iwata city hospital, and the research was conducted in accordance with the ethical principles stated by the Declaration of Helsinki. The requirement for obtaining informed consent was waived by the research ethics committee based on the retrospective design of this study. Instead, a detailed disclosure of this study contents was published on the website of the research ethics committee. Patient records/information was anonymized and de-identified prior to analysis.

### Data collection

The following patient information during hospitalization was documented: sex, age, type of cancer, chemotherapy regimen, performance status, hemoglobin, glycated hemoglobin, serum albumin, estimated glomerular filtration rate (eGFR), serum creatinine (SCr), mean blood pressure, body mass index (BMI), C-reactive protein, the dose of cisplatin, the volume of hydration after the cisplatin administration in the same day, concurrent radiation therapy, and drugs associated with kidney function such as nonsteroidal anti-inflammatory drugs, magnesium, mannitol, renin-angiotensin system inhibitor and organic cation transporter 2 inhibitor (histamine H2 receptor antagonist or proton pump inhibitor) [14–16].

### Nephrotoxicity evaluation

We used the changes of eGFR before and after cisplatin administration for the evaluation of nephrotoxicity. The lowest eGFR from day 3 to 14 was applied as the peak of kidney injury at acute phase:

$$Change\;of\;eGFR\;(\%)=\left[1-(\frac{eGFR\;at\;the\;peak\;of\;kidney\;injury}{eGFR\;just\;before\;cisplatin\;treatment})\right]\times 100$$

The eGFR was obtained using the Japanese GFR calculation formulas prepared by the Japanese Society of Nephrology as following [17]:

$$eGFR\;(mL/min/1.73\;m^{2})=194\times SCr^{-1.094\;}\times Age^{-0.287}\times 0.739\;(if\;female)$$

The definition of AKI was also referred to the criteria of the KDIGO classification based on the changes of SCr levels [18]:

Stage 1: increase in SCr > 0.3 mg/dL or 1.5 to 1.9 multiplied by baseline; Stage 2: 2.0 to 2.9 multiplied by baseline SCr; Stage 3: 3.0 or more multiplied by baseline SCr or increase in SCr > 4.0 mg/dL.

### Statistical analysis

Differences in categorical outcomes were evaluated using the Fisher’s exact test. Differences between two groups were assessed using an unpaired *t*-test or Mann-Whitney U test, as appropriate. All statistical analyses were performed using GraphPad Prism version 6 (GraphPad Software, San Diego, CA, USA). A p-value < 0.05 was accepted as statistically significant.

## Results

### Patient characteristics

A total of 34 patients were eligible for the analysis (Fig 1). The clinical characteristics and treatment methods of the patients were summarized in Table 1. The mean age of the patients was 65 (47–78) years old including 28 men (82%) and 6 women (18%). Most patients had a good performance status of 0–1. All patients received dexamethasone and 5-HT3 receptor antagonist as antiemetics. BMI was significantly lower in DPP-4 inhibitor group, compared to non-DPP-4 inhibitor group (P = 0.020), but other parameters were not different between 2 groups. In DPP-4 inhibitor group, 30% of patients had been prescribed anti-diabetic drugs which induce weight gain such as insulin, sulfonylurea and thiazolidine. On the other hand, 42.9% of patients had been prescribed such drugs in non-DPP4 inhibitor group. In DPP-4 inhibitor group, 40% of patients had been prescribed metformin which induces slight weight loss [19], while none of the patients had been prescribed metformin in non-DPP-4 inhibitor group.

**Table 1:** Patient characteristics just before cisplatin administration and other treatment methods in user or non-user of DPP-4 inhibitors.

Characteristics	DPP-4 inhibitor group	non-DPP-4 inhibitor group	P value
**Number of patients**	20	14	
**Male**; number (%)	17 (85)	11 (79)	0.827
**Age, years**; mean (range)	65 (49–73)	65 (47–78)	0.672
**Performance status**; number (%)			
0–1	19 (95)	13 (100)	1.000*^1^
2–4	1 (5)	0	
**Type of cancers**; number (%)			
Lung (NSCLC)	12 (60)	5 (36)	
Lung (SCLC)	0 (0)	1 (7)	
Tongue	1 (5)	1 (7)	
Esophagus	4 (20) *^2^	3 (21)	
Stomach	4 (20) *^2^	4 (29)	
**Hemoglobin (g/dl)**; mean (range)	12.4 (9.9–15.6)	12.2 (7.3–14.5)	0.931
**Glycated hemoglobin (%)**; mean (range)	7.4 (5.3–10.5) *^3^	7.1 (6.5–8.9) *^4^	0.452
**Serum albumin (g/dl)**; mean (range)	3.6 (2.3–4.5)	3.5 (2.1–4.2)	0.775
**eGFR (ml/min./1.73m**^**2**^**)**; mean (range)	91 (51–128)	85 (62–134)	0.397
**Serum creatinine (mg/dl)**; mean (range)	0.68 (0.49–1.11)	0.70 (0.51–0.93)	0.737
**Mean blood pressure (mmHg)**; mean (range)	88 (68–128)	85 (70–98)	0.599
**Body Mass Index**; mean (range)	20.8 (17.6–27.1)	23.2 (19.1–30.0)	0.020
**C-reactive protein (mg/dl)**; mean (range)	1.72 (0–8.50)	3.30 (0–20.9)	0.320
**Regimen**; number (%)			
Cisplatin + Pemetrexed	2 (10)	1 (7)	
Cisplatin + Pemetrexed + Bevacizumab	1 (5)	0	
Cisplatin + Pemetrexed + Pembrolizumab	1 (5)	0	
Cisplatin + Tegafur/gimeracil/oteracil	3 (15)	3 (21)	
Cisplatin + Docetaxel	3 (15)	1 (7)	
Cisplatin + Etoposide	2 (10)	2 (14)	
Cisplatin + 5-FU	4 (20)	3 (21)	
Cisplatin + Vinorelbine	1 (5)	1 (7)	
Cisplatin + Gemcitabine	0	1 (7)	
Cisplatin + Irinotecan	2 (10)	0	
Cisplatin + Trastuzumab	0	1 (7)	
Cisplatin only	1 (5)	1 (7)	
**Dose of cisplatin (mg/m**^**2**^**)**; mean (range)	70.6 (58–100)	70.5 (56–80)	0.967
**Hydration dose after cisplatin (ml)**; mean (range)	1467 (750–2500)	1613 (500–2000)	0.443
**Concurrent radiation therapy**; number (%)	8 (40)	8(57)	0.487
**Co-administrated NSAIDs**; number (%)	2 (10)	3 (21)	0.627
**Co-administrated magnesium sulfate**; number (%)	9 (45)	3 (21)	0.275
**Co-administrated mannitol**; number (%)	16 (80)	9 (64)	0.435
**Use of renin-angiotensin system inhibitor**; number (%)	4 (20)	2 (14)	1.000
**Use of organic cation transporter 2 inhibitor**; number (%)	5 (25)	6 (43)	0.458
**Other diabetic treatment**; number (%)			
Insulin	0	3 (21)	
Metformin	4 (20)	0	
Sulfonylurea	0	2 (14)	
Thiazolidine	0	1 (7)	
Glucagon-like peptite-1 agonist	0	0	
Glinide	0	0	
α-glucosidase inhibitor	0	1 (7)	
Sodium-glucose transport protein 2 inhibitor	0	1 (7)	
Metformin + insulin	2 (10)	0	
Metformin + sulfonylurea	1 (5)	0	
Sulfonylurea + thiazolidine	1 (5)	0	
Sulfonylurea + α-glucosidase inhibitor	1 (5)	0	
Metformin + sulfonylurea + α-glucosidase inhibitor	1 (5)	0	
No other treatment	10 (50)	6 (42)	

Abbreviation: eGFR, estimated glomerular filtration rate. NSCLC, non-small-cell lung carcinoma. NSAIDs, non-steroidal anti-inflammatory drugs.

*1: A case does not have information about Performance status.

*2: A case had double cancers.

*3: A case did not check the level near chemotherapy.

*4: Three cases did not check the level near chemotherapy.

### Evaluation of the renal parameters

**Fig 2:**
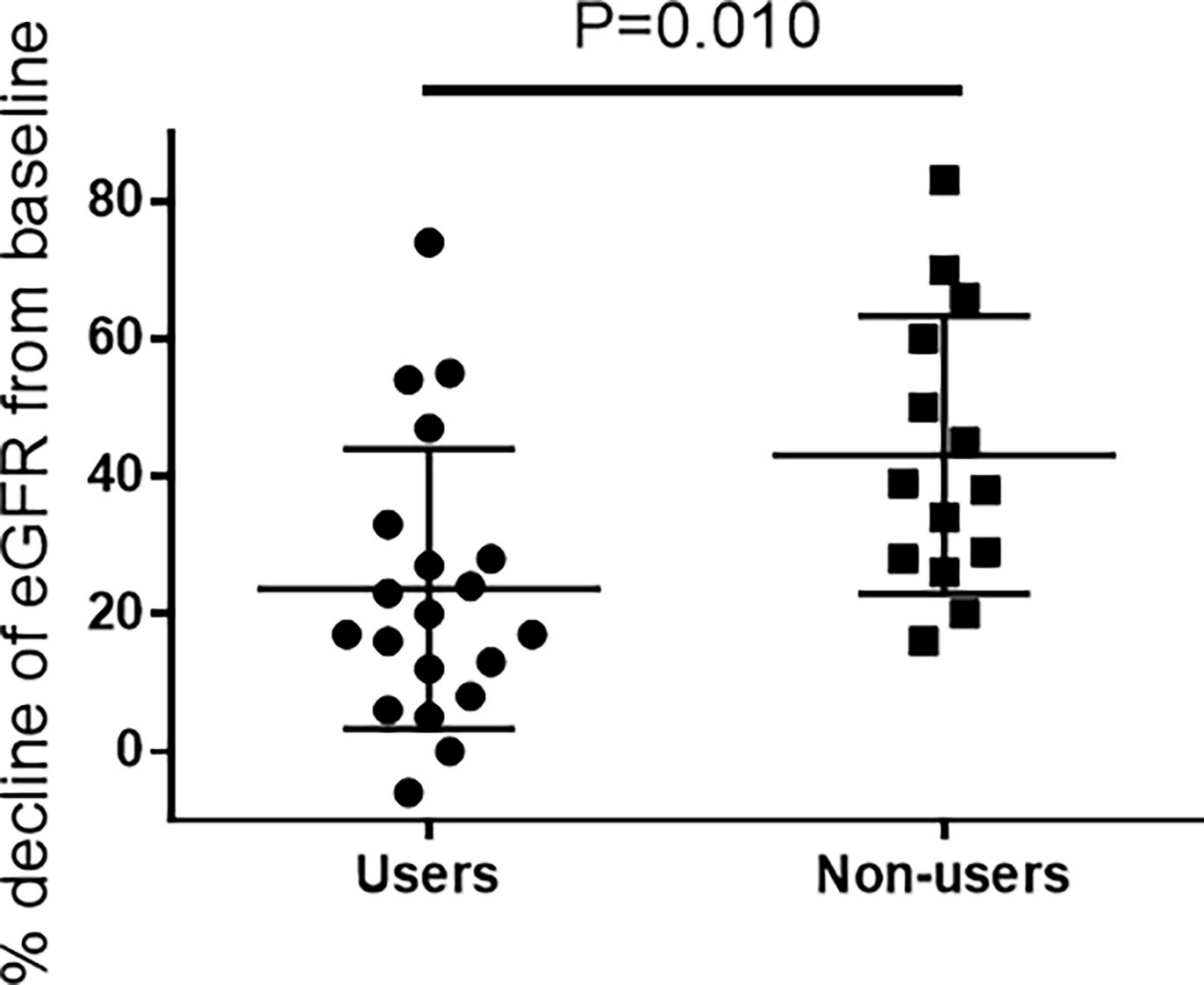
The change of eGFR at acute phase after cisplatin administration. An unpaired t-test was performed to compare the percentages of eGFR decline in users and non-users of DPP-4 inhibitors. Data are presented as mean ± SD.

The eGFR decline at acute phase was significantly less in DPP-4 inhibitor group, compared to non-DPP-4 inhibitor group (Fig 2 and Table 2; P = 0.010). Furthermore, the incidence of AKI was significantly less in DPP-4 inhibitor group (25% vs 64%, respectively; P = 0.026), suggesting that DPP-4 inhibitors can attenuate cisplatin-induced AKI (Table 2).

**Table 2:** Cisplatin nephrotoxicity in user or non-user of DPP-4 inhibitors.

Characteristics	DPP-4 inhibitor group	non-DPP-4 inhibitor group	P value
**% decline of eGFR**; mean (SD)	23.6 (20.3)	43.1 (20.1)	0.010
**AKI incidence**; number (%)	5 (25)	9 (64)	0.026
**AKI Stage**; number			
1	2	5	0.043
2	2	2	0.328
3	1	2	0.210

Abbreviations: eGFR, estimated glomerular filtration rate. AKI, acute kidney injury.

## Discussion

In the present study, we investigated whether DPP-4 inhibitors might affect AKI outcomes in diabetic-cancer patients treated with high-dose cisplatin. Our data demonstrate that (i) the kidney function in diabetic-cancer patients treated with cisplatin is significantly preserved by using DPP-4 inhibitors, and (ii) the incidence of AKI is reduced by these drugs. Katagiri and we have shown that DPP-4 inhibitors attenuated cisplatin-induced kidney injury in mice and rats, respectively [4, 5]. Katagiri and others have also revealed that the effect of DPP-4 inhibitor was mediated by glucagon-like peptide-1 (GLP-1) [4], suggesting that any medicines which increase the level of GLP-1 could prevent cisplatin-induced kidney injury. In other words, it suggests that any kind of DPP-4 inhibitors could attenuate cisplatin-induced kidney injury. In this regard, our data revealed for the first time that DPP-4 inhibitors had a potential to attenuate cisplatin-induced kidney injury in human patients. As there were no patients using GLP-1 receptor agonist in the present study, further studies to investigate whether GLP-1 receptor agonist also can attenuate cisplatin nephrotoxicity in human patients should be performed.

The mechanism of action could not be evaluated in this study. The pathophysiology of cisplatin nephrotoxicity is thought to be induced by multiple mechanisms such as proximal tubular injury, oxidative stress, inflammation, and vascular injury in the kidney [20]. The renoprotective effects of DPP-4 inhibitors on chronic kidney disease have been reported in several clinical trials, and recently reviewed by some researchers [21, 22]. Some of clinical trials have shown the reduction of albuminuria [23–26], but the improvement of kidney function has been limited [27]. The underlying mechanisms of the effect is thought to be mostly related to the reduction of oxidative stress and inflammation, resulting in endothelial protection [28]. DPP-4 inhibitors decreased pro-inflammatory cytokines such as tumor necrosis factor-α, interleukin-1 beta and interleukin-6 [29, 30], and increased anti-oxidants [31]. It has also been reported that DPP-4 inhibitors decreased oxidative stress and inflammation in the kidney after cisplatin administration in animal models [4, 5]. In addition, it has been reported that GLP-1 receptor agonists reduced oxidative stress and inflammation in the kidney in ischemia-reperfusion injury, and protected endothelial cells from oxidative stress-induced injury [32, 33]. Regarding tubular protection with DPP-4 inhibitors, to the best of our knowledge, there have been no reports in human patients yet. In in vitro experiments, we found that pretreatment of DPP-4 inhibitor or GLP-1 receptor agonist could not attenuate cisplatin cytotoxicity in proximal tubular cells (unpublished data), suggesting that the renoprotective effect of DPP-4 inhibitors/GLP-1 is not due to the direct protection of proximal tubular cells. DPP-4 cleaves many substrates. Of these DPP-4 substrates, stromal cell-derived factor (SDF)-1α has been reported to attenuate kidney injury induced by ischemia-reperfusion [34]. In in vitro experiment, we showed that treatment of SDF-1α decreased cisplatin cytotoxicity in mouse proximal tubular cells [5]. Hence, the increase of SDF-1α by DPP-4 inhibitors might contribute to the tubular protection in human patients. However, it should be noted that DPP-4 inhibitor did not change the plasma level of SDF-1α at least in mice model of cisplatin-induced AKI [4]. Additionally, it has been reported that GLP-1 increased heme oxygenase-1 [35], whose overexpression in proximal tubular cells inhibited cisplatin cytotoxicity [36]. Further studies are required to clarify the precise mechanism of DPP-4 inhibitors on cisplatin nephrotoxicity in human patients.

In our study, approximately 40% of patients who were treated with high-dose cisplatin experienced AKI. The incidence was consistent with the recent report which contained patients treated with similar dose of cisplatin [37]. On the other hand, it has been reported that diabetes mellitus may be a risk factor of cisplatin-induced AKI [38]. Our data show that the usage of DPP-4 inhibitors to diabetic-cancer patients could reduce the incidence of cisplatin-induced AKI from 64% to 25%, but its preventive measure is still insufficient. A variety of mechanisms have been reported to prevent cisplatin-induced AKI in pre-clinical studies [20]. Further clinical studies using the medicines which mediate these uninvestigated mechanisms are required to resolve cisplatin nephrotoxicity.

In this study, BMI in patients treated with DPP-4 inhibitors was lower, compared to those without DPP-4 inhibitors. It has been reported that DPP-4 inhibitors per se did not influence the body weight [39]. On the other hand, insulin, sulfonylurea and thiazolidine are known to induce weight gain [39]. On the contrary, it has been reported that metformin decreased body weight, and inhibited the weight gain by insulin and sulfonylurea [19, 40, 41]. In fact, 42.9% of the patients were prescribed insulin, sulfonylurea and thiazolidine in non-DPP-4 inhibitor group, but 30% of patients in DPP-4 inhibitor group. Furthermore, 40% of the patients were prescribed metformin in DPP-4 inhibitor group, but none of the patients in non-DPP-4 inhibitor group. Therefore, the lower BMI in DPP-4 inhibitor group could be caused by fewer use of insulin, sulfonylurea and thiazolidine and the use of metformin.

Several limitations in this study should be described. Firstly, this study was a retrospective design with small sample size. Secondly, cisplatin was combined with other various chemotherapeutic agents in most of the patients. Thirdly, the effect of DPP-4 inhibitors on the tumor response was difficult to evaluate because patients in this study included a variety types of cancer with different stages. Of note, it has been reported that anti-DPP-4 monoclonal antibody or vildagliptin, a DPP-4 inhibitor, suppressed cancer cell growth and metastasis in both in vitro and in vivo studies [42–44]. A prospective/randomized study with greater number of patients, a uniform protocol and adjustment of patients’ background are needed to confirm the beneficial effects of DPP-4 inhibitors. In fact, Baek et al. opened a study protocol of randomized double-blind, placebo-controlled trial to examine the effect of DPP-4 inhibitor in cancer patients [12], but the results are not yet reported.

## Conclusion

The use of DPP-4 inhibitors could improve the renal outcome after cisplatin treatment in diabetic-cancer patients. Therefore, the use of DPP-4 inhibitors may be one of supportive therapies for cancer patients treated with high-dose cisplatin.

## Supporting information

S1 Fig The raw data of eGFR change, cisplatin dose and incidence of AKI. The raw data are shown separately.
(PZF)

S2 Fig The raw data of Table 1. The raw data of each parameter are shown separately.
(PZF)
